# T132. ASSESSMENT OF CROSS-NATIONAL EQUIVALENCE OF THE COMMUNITY ASSESSMENT OF PSYCHIC EXPERIENCES (CAPE)

**DOI:** 10.1093/schbul/sby016.408

**Published:** 2018-04-01

**Authors:** Baptiste Pignon, Hugo Peyre, Aziz Ferchiou, Jim van Os, Bart P F Rutten, Robin M Murray, Craig Morgan, Marion Leboyer, Franck Schürhoff, Andrei Szöke

**Affiliations:** 1 INSERM; 2 Robert Debré Hospital; 3 University Medical Center Utrecht; 4 King’s College London

## Abstract

**Background:**

The Community Assessment of Psychic Experiences (CAPE) is a self-report questionnaire that has been developed to measure the dimensions of psychosis in the general population. The cross-national equivalence of a questionnaire allows the comparability of a scale across different populations in different countries, i.e., using different versions of the scale according to the considered language. In this study, our aim was to investigate the equivalence of the CAPE across different countries.

**Methods:**

Data were drawn from the European Union Gene-Environment Interaction (EU-GEI) study. Participants (incident case of psychotic disorder, controls and siblings of cases) were recruited across in six countries: United Kingdom, the Netherlands, Italy, Spain, Brazil and France. To analyse the cross-national equivalence of the dichotomised version of the CAPE, we used the multigroup categorical confirmatory factory analysis (MCCFA). The cross-national equivalence can be stated after the establishment of three invariances characterised by increased constraints: the configural invariance, the metric invariance and the scalar invariance across the multiples groups.

**Results:**

The configural invariance model fits well, providing evidence for identical factor structure across countries. The assumption that factor loadings are identical across countries is granted based on the negligible change in the fit indices in the metric invariance model. Moreover, the fit indices suggest that the CAPE shows scalar invariance across countries.

**Discussion:**

These findings suggest that comparisons across countries of factor and observed means of the CAPE are possible. Thus, differences observed in scores between samples from different countries can be considered as different levels of psychosis.

